# Effect of temperature on betacyanins synthesis and the transcriptome of *Suaeda salsa*

**DOI:** 10.3389/fpls.2023.1203089

**Published:** 2023-06-26

**Authors:** Min Li, Peimin He, Zitao Zhao, Jinlin Liu, Hongtao Liu, Shaozu Ma, Yifei Shen, Bin Li

**Affiliations:** ^1^ School of Marine Ecology and Environment, Shanghai Ocean University, Shanghai, China; ^2^ Engineering Research Center for Water Environment Ecology in Shanghai, Shanghai, China; ^3^ Key Laboratory of Exploration and Utilization of Aquatic Genetic Resources, Ministry of Education, Shanghai Ocean University, Shanghai, China

**Keywords:** *Suaeda salsa*, transcriptome, betacyanins, temperature, coastal wetlands

## Abstract

**Introduction:**

*Suaeda salsa* (Linn.) Pall. is an important tourist resource and ecological restoration species in coastal wetlands. Environmental factors such as low temperature, darkness, phytohormone, salt stress and seawater flflooding, and light can induce betalain synthesis in *S. salsa*, which plays an important role in plant adaptation to abiotic stress processes and in shaping the beautiful “red beach” landscape.

**Methods:**

In this study, Illumina sequencing was used to profifile the transcriptome sequence (RNA-Seq) of *S. salsa* leaves at different temperatures (5° C, 10°C, 15°C, 20°C, 25°C, and 30°C) and to validate differentially expressed genes (DEGs) indicated by real-time PCR (RT-qPCR).

**Results:**

The betacyanin content was highest in *S. salsa* leaves at 15°C. Transcription group data showed that compared to the control group (15°C), the “betacyanin biosynthesis pathway” was signifificantly enriched in the fifive different temperature groups. KEGG analysis showed that the DEGs were mainly involved in pathways of phenylpropanoid biosynthesis, carbon fifixation in photosynthetic organisms, flflavonoid biosynthesis, and betacyanin biosynthesis. Among the key enzymes involved in biosynthesis of betacyanin, genes for tyrosinase, CYP76AD1 and 4,5-DOPA dioxygenase were signifificantly upregulated and most abundantly expressed at 15°C. It is possible that the gene for betacyanin synthesis from *S. salsa* is primarily regulated by the MYB1R1 and MYB1 transcription factor. Four DEGs were randomly selected for quantitative PCR analysis, and DEG expression was generally consistent with the RNA-Seq data, verifying the validity of the transcriptome sequencing data.

**Discussion:**

Relative to other temperatures, 15°C was optimum for *S. salsa* betacyanin synthesis, and this provides a theoretical reference for coastal wetland ecological remediation, reveals mechanisms of *S. salsa* discoloration, and further mines its potential application for landscape vegetation.

## Introduction

In recent years, under the influence of various factors such as global climate anomalies, intensified human activities, and sea level rise, the area of global coastal wetlands has decreased sharply, and the ecological environment and wetland animal and plant diversity have been damaged to varying degrees (Zhang, 2022). Since the 1950s, the total area of coastal wetlands in China has decreased by more than 50%, including a 59% reduction in the area of salt marsh vegetation (Yang et al., 2006; Gu et al., 2018). There have been many studies on ecological restoration measures for *Suaeda salsa* (Linn.) Pall. communities in degraded saline lands (Sui et al., 2013), but the mechanism responsible for betacyanin change remains unclear (Jia et al., 2018).

*Suaeda salsa* is an annual halophytic herb, belonging to the Amaranthaceae, Caryophyllaceae (Liu et al., 2022). Planting *S. salsa* can effectively reduce soil salinity and play an important role in wetland purification and ecological restoration of saline and alkaline lands (Cong et al., 2013). *Suaeda salsa* contains betacyanins, which are very important natural plant pigments with high antioxidant and antibacterial activities (Gengatharan et al., 2015), and are widely used in food processing, medicine, pharmacy, and other fields (Ninfali et al., 2017). Due to the presence of betacyanins, *S. salsa* plants are bright red. In the deep autumn, large areas of *S. salsa* form a “red beach,” which is of great ornamental value in intertidal mudflat areas. However, there have been few studies on the landscape ecology of betacyanins, particularly at the molecular level. Therefore, exploring the synthesis and regulation mechanism of betacyanins in *S. salsa* can provide theoretical reference for further exploring its potential application value as landscape vegetation.

Previous studies showed three main pathways for the betacyanin synthesis from *S. salsa*: betalain and cyclo-DOPA react to form betanin, glycosylated cycloDOPA, and the tyrosine-betaxanthinc pathways. Among them, the betalain and cyclo-DOPA react to form betanin pathway has been most studied (Jia et al., 2018). Tyrosinase, 4,5-DOPA-extradiol-dioxygenase (DOD), 5-O-glucosyl transferase (5GT) and CYP76AD1 encoding a novel cytochrome P450 are the four key enzymes in betacyanin biosynthesis, with their activities positively correlated with *S. salsa* betacyanin content (Gandia and Garcia, 2019). Research on betacyanin biosynthesis and regulation has mainly focused on the aldosine-cycloDOPA pathway and the three key enzymes mentioned above.

Light, temperature, and salinity are the most critical factors affecting betacyanin biosynthesis. It has not yet been possible to regulate cultivation of all-red *S. salsa* plants throughout their life cycle under laboratory conditions. Darkness, high salt, and low temperature are beneficial to betacyanin accumulation in *S. salsa*. Dark treatment during seed germination is important for triggering betacyanin accumulation in *S. salsa* (Gandia-Herrero et al., 2005). At the same time, tyrosinase activity can only be detected in *S. salsa* cultivated in darkness (Wang, 2009). Zheng et al. (2015) studied the effect of different light qualities on betacyanin synthesis of *Amaranthus tricolor* L., and showed that blue light was more favorable than red and green light. Soil salinity has a significant impact on composition of pigments in *S. salsa* leaves, and there is a positive correlation between soil Na^+^ content and betacyanin content in *S. salsa* leaves (Zheng et al., 2015). However, single-factor salt treatment is not conducive to betacyanin accumulation in *S. salsa*, and there is a significant negative correlation between salt concentration and betacyanin content (Li et al., 2018). Combined salt and nitrogen treatment can significantly increase *S. salsa* betacyanin content (Zhao et al., 2020).

Temperature has a significant impact on betacyanin stability, and high temperature can inhibit the betacyanin synthesis in *A. tricolor* and *Beta vulgaris* L.; low temperature is not conducive to betacyanin degradation (Elliott, 1979; Cui, 2020; Sokolova et al., 2022). Ruan (2008) cloned the promoter *Ss DODA* of the gene for 4,5-DOPA dioxygenase from the *S. salsa* genome using TAIL-PCR technology, and showed that this promoter contained a low-temperature response element (LTR; CCGAAA). Further studies showed that low temperature is beneficial for upregulation of the gene for 4,5-DOPA dioxygenase in *S. salsa*, and promotes betacyanin biosynthesis in *S. salsa* (Wang, 2011). The mechanism of the effect of different temperatures on betacyanin synthesis and metabolism in *S. salsa* needs further study.

This study employed transcriptome technology to explore the molecular response mechanism of *S. salsa* betacyanin synthesis to external temperature (using a temperature gradient of 5°C, 10°C, 15°C, 20°C, 25°C, and 30°C), and to reveal the optimal temperature for synthesis. The purpose is to provide a theoretical basis for in-depth discussion of the coloration mechanism of *S. salsa*, and to provide a reference for restoration of the *S. salsa* community in coastal wetlands of northern China and to add a touch of “red beach.”

## Materials and methods

### Plant sample

The *S. salsa* seeds used in this experiment were purchased from Suqian City, Jiangsu Province in December 2021. In January 2022, full *S. salsa* black seeds were selected and sterilized with 1% potassium permanganate solution for 20 min, followed by repeated rinsing with sterile water. After soaking for 24 h, they were sown in a flowerpot with holes at the bottom, cultured under conditions of diurnal temperature of 25/20°C (light intensity 600 μmol m^−2^ s^−1^, 14-/10-h light/dark cycle), and irrigated daily with half-strength Hoagland nutrient solution containing 100 mmol·l^−1^ NaCl. When the sixth pair of leaves of *S. salsa* grew steadily, seedlings with good and consistent growth condition were selected and placed in a light incubator at 5°C, 10°C, 15°C, 20°C, 25°C, and 30°C for a 4-day culture experiment, with three parallel sets for each temperature gradient. After culture, the same part of leaves of three parallel plants in each temperature group were combined into one sample, and quickly rinsed with ultra-pure water to remove surface sludge. Excess water was absorbed with filter paper, and the leaves were cut into 0.5 cm × 0.5 cm pieces, placed in a 1.5-ml enzyme-free centrifuge tube, quickly frozen in liquid nitrogen, and stored in a refrigerator at −80°C for subsequent analysis.

### Determination of betacyanin content

Determination of betacyanin content of *S. salsa* was based on the method of Li (2021), with appropriate improvements. Leaves (0.2 g) were taken from the same part of different treatments and ground under liquid nitrogen, then extracted with 20 ml of methanol for 30 min, and centrifuged at 4°C and 11,000 g for 10 min. The supernatant was discarded and the residue was extracted three times with methanol. The precipitate was extracted with 20 ml of pure water for 30 min, centrifuged at 4°C and 16000 g for 10 min, and the supernatant was taken. This operation was repeated three times until the betacyanins were completely extracted. Finally, the collected pure water extract was brought to a constant volume. The absorbance value at 538 nm was measured using an ultraviolet spectrophotometer, and the relative betacyanin content was expressed using OD538. Statistical calculations and analysis of variance were performed using Excel 2019 for the data.

### Library preparation and illumina hiseq X ten sequencing

Total RNA in *S. salsa* leaves was extracted from the six temperature gradient groups. The RNA library preparation and transcriptome sequencing were completed by Shanghai Majorbio Bio-Pharm Technology Co., Ltd (www.majorbio.com). The samples were divided into six groups: Ss5, Ss10, Ss15, Ss20, Ss25, and Ss30 for 5°C, 10°C, 15°C, 20°C, 25°C, and 30°C, respectively. Based on the Illumina Novaseq 6000 sequencing platform, a library was constructed using the Illumina TruseqTM RNA sample prep kit. A Nanodrop2000 was used to determine the concentration and purity of the extracted RNA, agarose gel electrophoresis was used to detect the integrity of RNA, and an Agilent 2100 was used to determine the RIN value. Oligo dT was used to enrich mRNA, under the action of reverse transcriptase, with six-base random primers added to invert the segmented mRNA into cDNA, then connected to the adapter, and finally sequenced on the Illumina platform, to compare the sequencing data and assembly results. Differential gene expression analysis was performed using DEseq2. In order to understand the molecular function of differentially expressed genes (DEGs) or the metabolic pathways and biological processes involved, GO (Gene Ontology) and KEGG (Kyoto Encyclopedia of Genes and Genome) enrichment analysis were conducted (Khorramdelazad et al., 2018).

### Quantitative real-time-PCR verification

To verify the transcriptome sequencing analysis results, four DEGs (two upregulated and two downregulated) were randomly selected from two differential groups, Ss15vsSs20 (i.e. Ss15 vs. Ss20) and Ss15vsSs25, for qRT-PCR detection. **Table 1** shows the primers designed for five genes. Referring to the method of Nolan et al. (2006), RNA was extracted and the actin gene family *ActinF* was selected as the internal reference gene. The cDNA was synthesized by reverse transcription using HiScript Q RT SuperMix for qPCR (+gDNA wiper), ChamQ SYBR Color qPCR Master Mix (2X) was selected as the quantitative PCR reagent, and RT-qPCR analysis was performed using an ABI7300 type fluorescence quantitative PCR instrument (Applied Biosystems, USA), in accordance with the manufacturer’s instructions. The 2^−ΔΔCT^ method was used for experimental data analysis.

**Table 1:** The primer sequences used for qRT-PCR.

Gene id	Sequence (5′-3′)	Product size (bp)
TRINITY_DN24971_c0_g1	F:AGCAGGCAGTCAAGGAGTA R:AAGAAGCCTAACCAAGCAA	252
TRINITY_DN1758_c0_g1	F:GAGCCTGAACCAGTAAGAA R:GAATAATAAGCCCATAACCT	268
TRINITY_DN14253_c0_g1	F:GTTCGTCGGATCTCACTT R:GTTCCCACCACATTTGTC	255
TRINITY_DN4658_c0_g1	F:TAGGACAGTTGCCAAAGGA R:CACCACCACAATCATCAGC	189
Actin F	F:GCTCTACCCCATGCAATCCT R:TGCTCTTGGCAGTCTCTGATT	190

### Phylogenetic tree construction

Using the NCBI BLAST network server, protein sequences were identified and amino acid sequences of the same family in the same or different species were found and retrieved from GenBank. Phylogenetic trees for analysis of TYRs, UGTs, CYP450s, DODAs were generated using MEGA11 software (Tamura et al., 2021). Node support was assessed using a bootstrap procedure based on 1000 replicates.

## Results

### Temperature and betacyanin content

**Figure 1:**
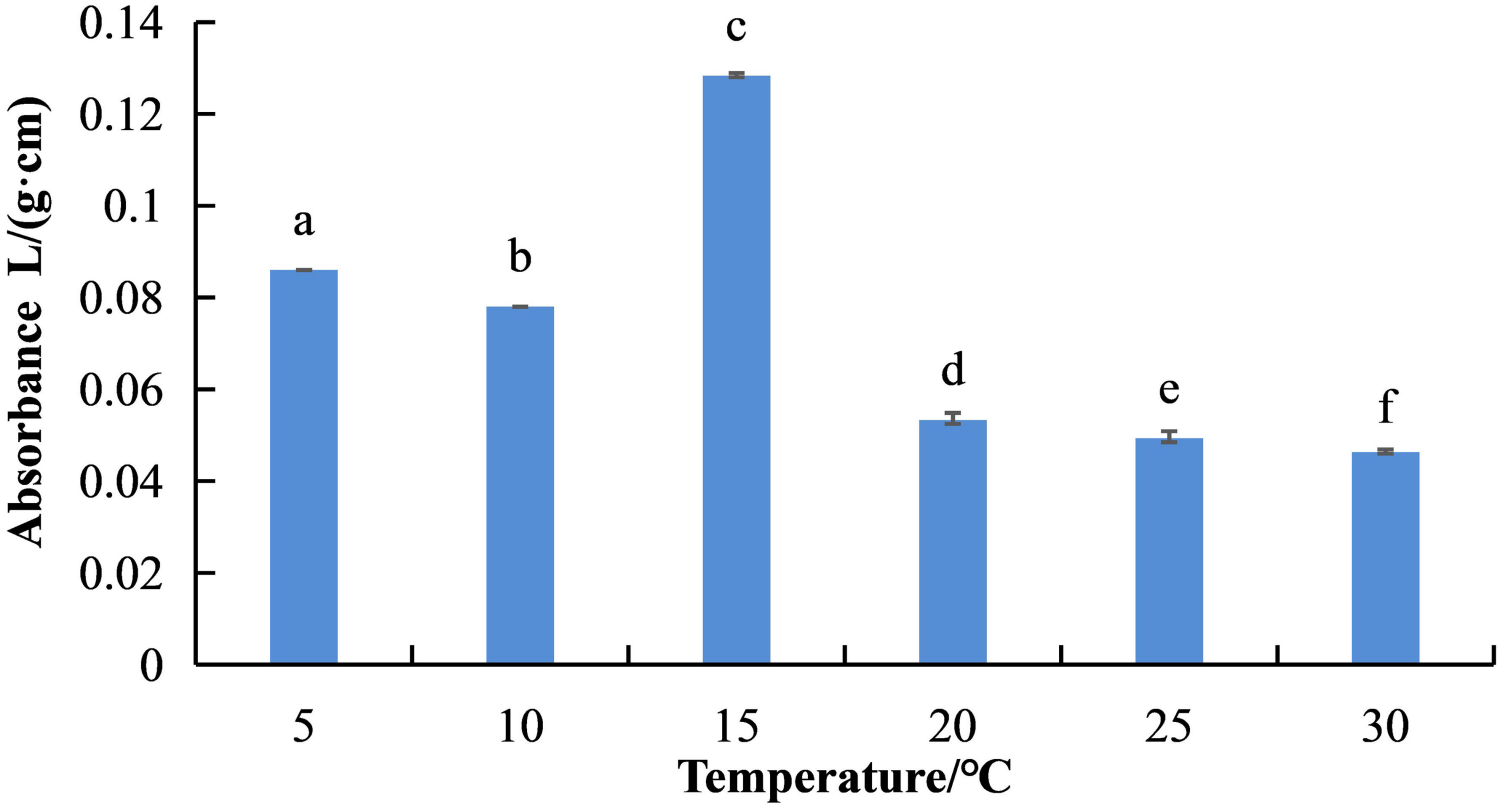
Differences in betacyanin content in *S. salsa* leaves under different temperature conditions. Lowercase letters indicate differences between groups under different temperature treatment conditions (*P* < 0.05). The error bars show std error.

Temperature had a significant impact on *S. salsa* betacyanin biosynthesis (**Figure 1**). At 15°C, the relative content of betacyanin in *S. salsa* leaves was the highest (absorbance value 0.1283), followed by 5°C (0.086) and 10°C (0.078). As temperature rose above 15°C, the betacyanin content decreased significantly (*P* < 0.05) in the following order: 20°C (absorbance value 0.053) > 25°C (0.049) > 30°C (0.046).

### Transcriptome sequencing analysis

To further explore the differences in gene expression in *S. salsa* leaves under different temperature conditions, transcriptome sequencing was performed on leaves at the same location from the six temperature groups. After RNA extraction and library construction, transcriptome sequencing was performed based on the Illumina platform. Transcriptome analysis of six samples was completed, and a total of 41.51Gb of clean data were obtained. Clean data of each sample exceeded 6.46 Gb, and the base percentage of Q30 was above 94.21% (**Table 2**). Trinity was used to reassemble all sample clean data from scratch, and to optimize and evaluate the assembly results. The number of unigenes assembled was 56,156, the number of transcripts was 93,108, and the average length of N50 was 1695 bp (**Table 3**). The clean reads of each sample were compared with the reference sequence obtained by Trinity assembly to obtain the mapping results for each sample. The analysis and comparison rates for the six samples were in the range of 81.84–84.87% (**Table 4**). The above results indicate that the transcriptome data obtained in this experiment were reliable and met the requirements for subsequent quantitative analysis of gene expression.

**Table 2:** Sequencing data statistics. Raw reads and raw bases represent the total number of entries and total data volume of the original sequencing data, respectively.

Sample	Raw reads	Raw bases	Clean reads	Clean bases	Error rate(%)	Q20 (%)	Q30 (%)	GC content(%)
SS30	45453016	6863405416	43912098	6460833761	0.0246	98.12	94.56	43.29
SS25	45773734	6911833834	44109872	6502311618	0.025	97.98	94.21	42.21
SS20	49617062	7492176362	48291196	7066224480	0.0243	98.26	94.89	43.55
SS15	52026972	7856072772	50591722	7225661138	0.0241	98.32	95.07	43.69
SS10	48275408	7289586608	47062338	6927938593	0.0247	98.13	94.47	43.89
SS5	50912970	7687858470	50140758	7322461426	0.0241	98.38	95.01	43.7

**Table 3:** Result of the *de novo* transcriptome assembly performed with Trinity.

Type	Unigene	Transcript
Total number	56156	93108
Total base	49676887	94984784
Largest length (bp)	14516	14516
Smallest length (bp)	201	201
Average length (bp)	884.62	1020.16
N50 length (bp)	1695	1728
E90N50 length (bp)	2496	2204
Fragment mapped percent(%)	71.886	85.743
GC percent (%)	38.92	38.93
TransRate score	0.29625	0.35641
BUSCO score	C:74.0%[S:72.8%;D:1.2%]	C:86.8%[S:52.6%;D:34.2%]

**Table 4:** Comparison of sequencing data and assembly results.

Sample	Clean reads	Mapped reads	Mapped ratio
SS30	21956049	18034784	82.14%
SS25	22054936	18601353	84.34%
SS20	24145598	19759816	81.84%
SS15	25295861	21005473	83.04%
SS10	23531169	19839561	84.31%
SS5	25070379	21277521	84.87%

### DEG analysis

**Figure 2:**
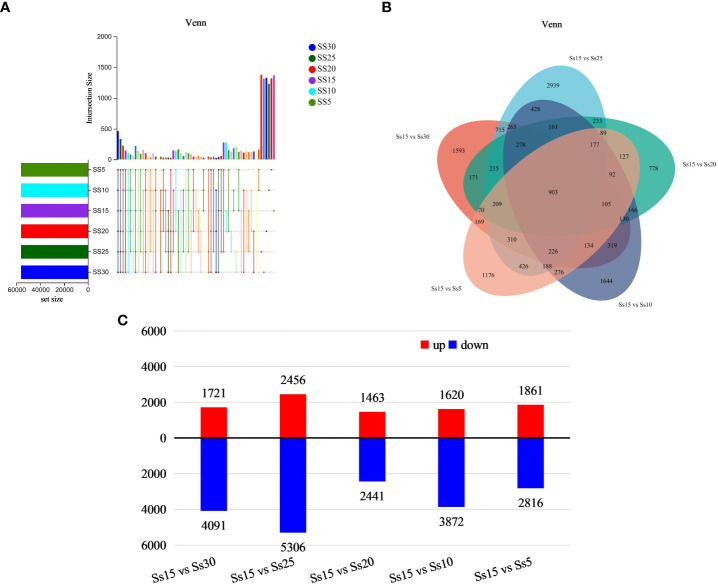
Differentially expressed gene (DEG) analysis. **(A)** Venn diagram of comparison of DEGs. **(B)** DEGs in Ss15vsSs30, Ss15vsSs25, Ss15vsSs20, Ss15vsSs10, and Ss15vsSs5. **(C)** Number of up-regulated and down-regulated genes in five different groups.

Based on the expression matrix, inter-sample Venn analysis was performed to obtain co-expression and specific expression genes between samples (**Figure 2A**). There were 463 co-expressed genes in the six samples, with 1311, 1378, 1330, 1370, 1234, and 1319 genes specifically expressed in the Ss30, Ss25, Ss20, Ss15, Ss10, and Ss5 samples, respectively.

Sample Ss15 was used as the control group, and samples Ss30, SS25, SS15, SS10, and SS5 as the experimental groups. Quantitative analysis of gene expression level was conducted using expression quantitative software RSEM, with the quantitative indicator being FPKM. The FPKM values of the six samples ranged within 0–25,495.38, with an average value of 15.19. The DEGseq software was used for inter-group differential expression analysis, with the parameters set as *P*-adjust < 0.01 and |log_2_FC| ≥ 2. A total of 903 DEGs were found in the five differential analysis groups (**Figure 2B**). Compared with the control group (15°C), there were 5812 DEGs at 30°C, including 1721 upregulated and 4091 downregulated; 7762 DEGs at 25°C, with 2456 upregulated and 5306 downregulated; 3094 DEGs at 20°C, including 1463 upregulated and 2441 downregulated; 5492 DEGs at 10°C, including 1620 upregulated and 3872 downregulated; and 4677 DEGs at 5°C, including 1861 upregulated and 2816 downregulated (**Figure 2C**).

### qRT-PCR verification

**Figure 3:**
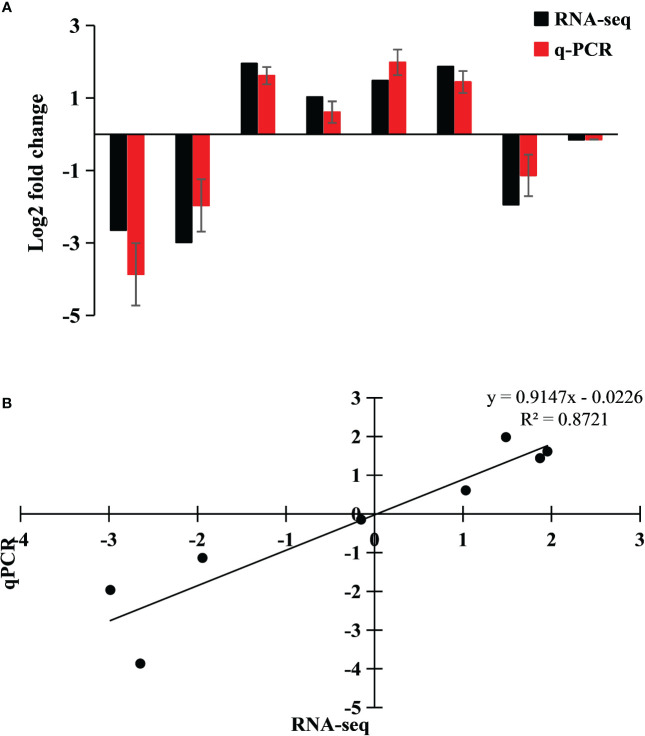
Expression of four differentially expressed genes (DEGs) in the transcriptome, verified by qRT-PCR. Fold changes are shown to verify the RNA-seq results. A **(A)** Genes used for validation from left to right: TRINITY_DN24971_c0_g1, TRINITY_DN1758_c0_g1, TRINITY_DN14253_c0_g1, and TRINITY_DN4658_c0_g1. **(B)** Linear regression fitting of RNA-seq and qRT-PCR DEGs.

In order to verify the accuracy of RNA-seq detection, using Ss15 as a control group, four DEGs (two upregulated and two downregulated) were randomly selected from the two differential groups of Ss15vsSs20 and Ss15vsSs25 for qRT-PCR (**Figure 3A**). Using RNA-seq and qRT-PCR as the abscissa and ordinate coordinates, respectively, the results were linearly regressed to fit the eight relative expression data of the four genes, with R^2 = ^0.8721 (**Figure 3B**), indicating that the expression patterns of the four genes were basically consistent with the RNA-seq results, further illustrating the reliability of transcriptome data.

### GO enrichment analysis of DEGs

**Figure 4:**
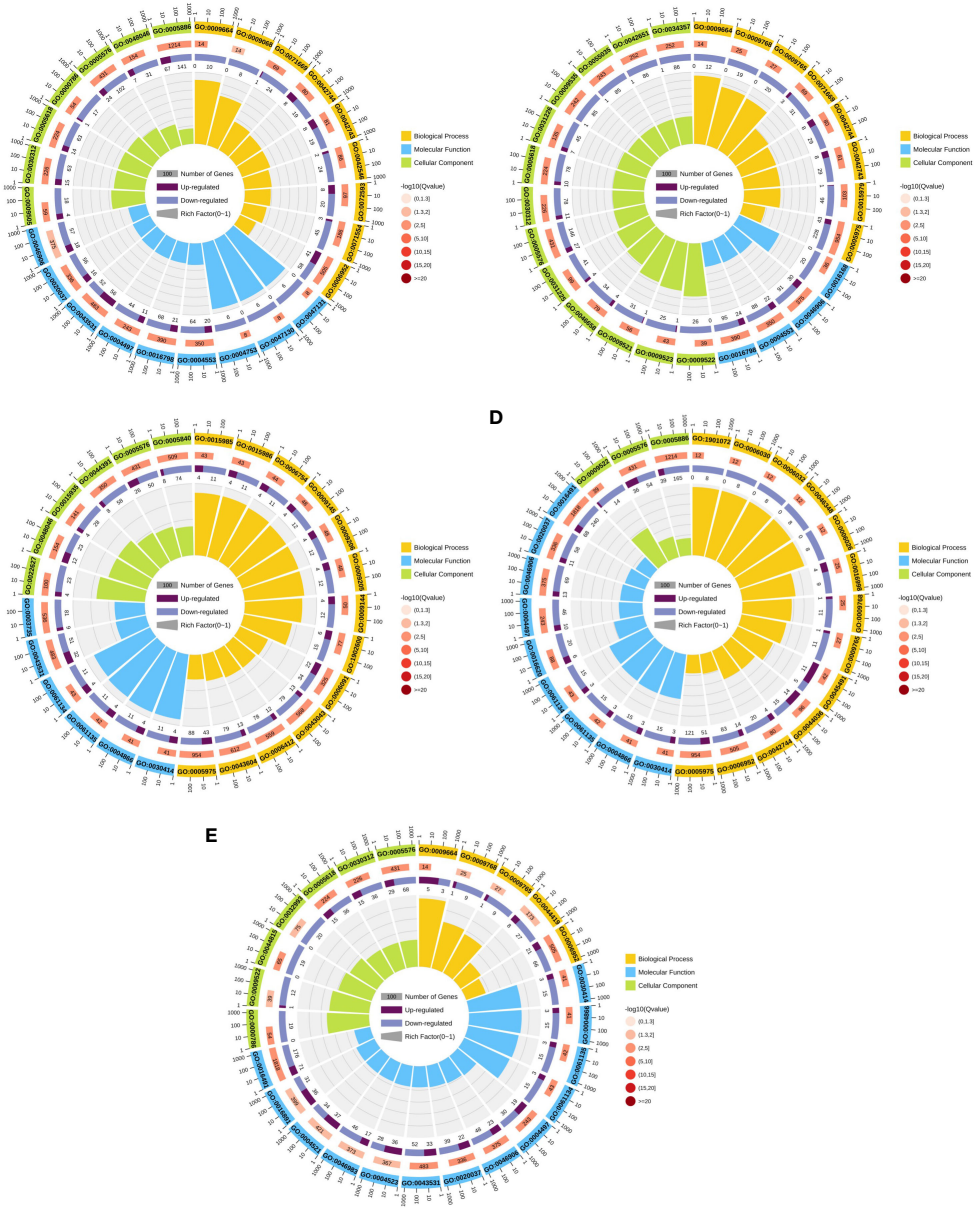
Gene ontology (GO) annotation analysis of DEGs in pairwise comparisons between the control (SS15) and other temperature treatments: **(A)** SS15vsSS30, **(B)** SS15vsSS25, **(C)** SS15vsSS20, **(D)** SS15vsSS10, and **(E)** SS15vsSS5. First circle: enriched classification, with a coordinate scale for the number of genes outside the circle. Different colors represent different classifications. Second circle: the number of the classification in the background gene and the Q or P value. The more genes, the longer the bars. The smaller the value, the redder the color. Third circle: bar graph of the proportion of up–down genes, deep purple represents an upregulated gene proportion, light purple represents the proportion of downregulated genes, and specific values are displayed below. Fourth circle: RichFactor values for each category (the number of foreground genes divided by the number of background genes in this classification), and each small grid of the background guides represents 0.1.

To further predict the function of DEGs, GO functional enrichment was used to analyze the DEGs in five groups: Ss15vsSs30, Ss15vsSs25, Ss15vsSs20, Ss15vsSs10, and Ss15vsSs5. The DEGs of the five differential comparison groups were basically annotated to the same GO category (**Figure 4**). Among the biological process categories, the three most representative GO categories were “defense response (GO:0006952),” “carbohydrate metabolic process (GO:0005975),” and “transport activity.” The category of molecular function mainly included “oxidoreductase activity,” “endopeptidase regulator activity (GO:0061135),” and “peptidase inhibitor activity (GO:0030414).” The classification of cell groups was mainly “cell part,” “membrane part,” and “organelle” (*P* < 0.05).

**Figure 4C** shows that Ss15vsSs20 significantly enriched the top 25 GO terms in three major categories: molecular function, cell composition, and biological process. With Q-value ≤ 0.05 as the screening condition, a total of 63 GO items were extremely significant enriched. In the biological process category, DEGs in the “purine nucleoside triphosphate biosynthetic process (GO:0009145),” “purine ribonucleoside triphosphate biosynthetic process (GO:0009206),” “energy coupled proton transport, down electrochemical gradient (GO:0015985),” “defense response (GO:0006952)”and other GOs terms were significantly enriched, and these terms enriched more down-regulation genes than up-regulation genes. In the cell composition category, our results showed that “cytosolic small ribosomal subunit (GO:0022627),” “ribosomal subunit (GO:0044391),” “apoplast (GO:0048046),” “extracellular region (GO:0005576),” were significantly enriched. In the molecular function, DEGs in the “endopeptidase inhibitor activity (GO:0004866),” “peptidase inhibitor activity (GO:0030414)”“endopeptidase regulator activity (GO:0061135),” “oxidoreductase activity (GO:0016491),” “monooxygenase activity (GO:0004497) were significantly enriched (**Table S1**).

### Biosynthetic pathway enrichment analysis

**Figure 5:**
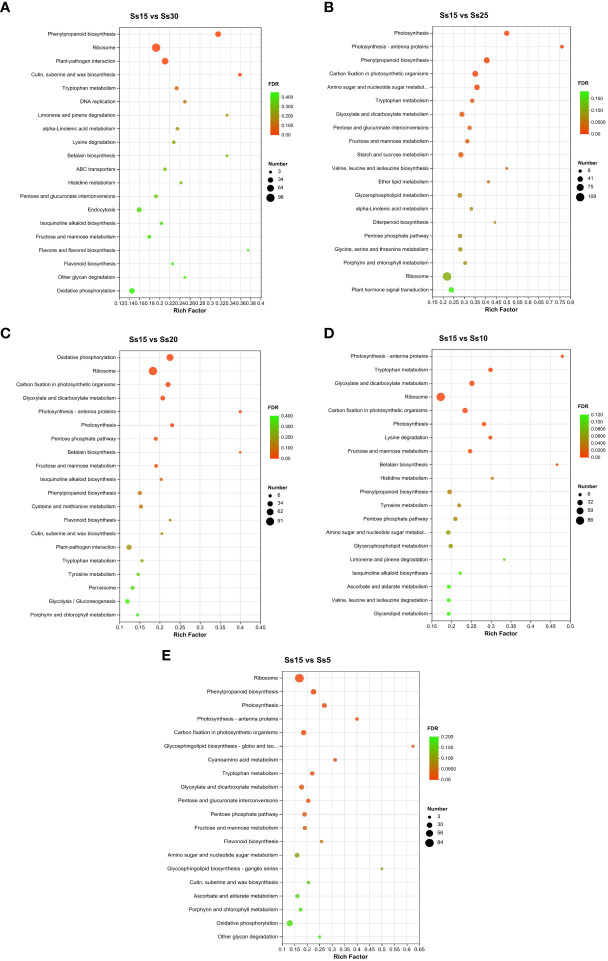
Kyoto Encyclopedia of Genes and Genomes (KEGG) enrichment analysis of DEGs in pairwise comparisons between the control (Ss15) and other temperature treatments: **(A)** Ss15vsSs30, **(B)** SS15vsSS25, **(C)** Ss15vsSs20, **(D)** Ss15vsSs10, and **(E)** Ss15vsSs5.

The DEG expression involved in biological pathways can be studied using KEGG to provide a developmental pathway network. Twenty KEGG pathways with the highest enrichment (TOP20) were selected from the five differential groups: Ss15vsSs30, Ss15vsSs25, Ss15vsSs20, Ss15vsSs10, and Ss15vsSs5 (**Figure 5**). The betacyanin biosynthesis pathway was significantly enriched in the five groups, among which it was significantly enriched to TOP20 in three groups (Ss15vsSs30, Ss15vsSs20, and Ss15vsSs10), with the most significant enrichment in Ss15vsSs20. The other two differential groups were enriched to the 21st (Ss15vsSs5) and 38th positions (Ss15vsSs25), consistent with the change trend of the betacyanin content results. The other significantly enriched pathways were successively “phenylpropane biosynthesis,” “carbon fixation in photosynthetic organisms,” “dicarboxylic acid metabolism,” “photosynthesis antenna protein,” “fructose and mannose metabolism,” “amino sugar and nucleotide sugar metabolism,” “ribosome,” and “flavonoid biosynthesis.”

**Figure 6:**
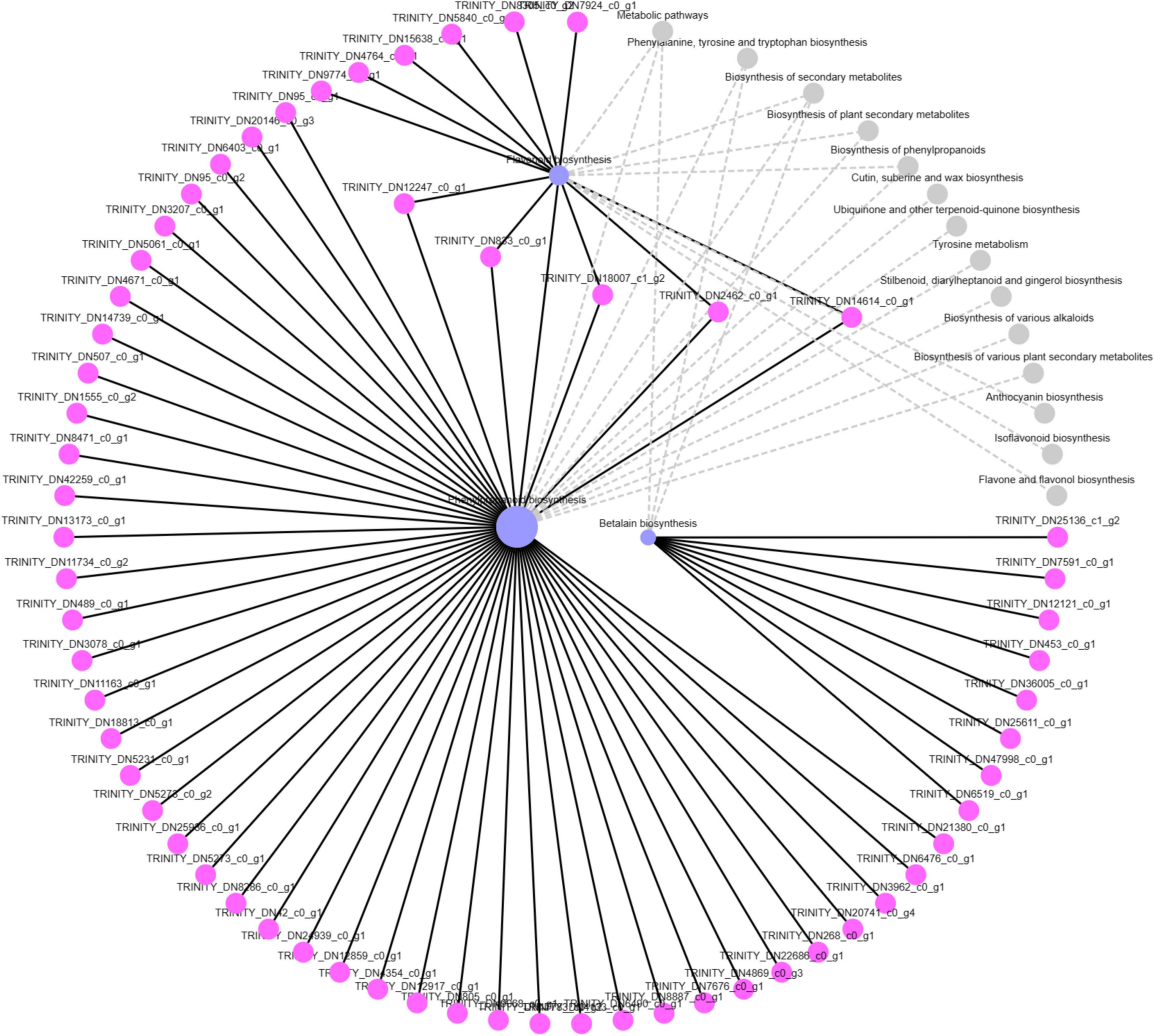
KEGG senior network chart of phenylpropanoid, flavonoid, and betalain biosyntheses.

To identify the function of DEGs enriched in the “biosynthesis of betanin” pathway, software Goatools was used to annotate the genes in this gene set (**Figure 6**). The results showed that the functional DEGs enriched in this pathway were mainly those for tyrosinase and 4,5-DOPA dioxygenase (predicted: tyrosine decarboxylase 1, 4,5-DOPA dioxygenase extradiol, tyrosinase-like protein orsC, and tyrosine/DOPA decarbox).

Among them, predicted: tyrosine decarboxylase 1 was annotated in *Beta* vulgaris subsp. Vulgaris (Gene ID: TRINITY_DN25136_c1_g2), 4,5-DOPA dioxygenase extradiol was annotated in *S. salsa* (Gene ID: TRINITY_DN12121_c0_g1), tyrosinase like protein orsC was annotated in *Quercus* suber (Gene ID: TRINITY_DN36005_c0_g1), tyrosine/DOPA decarboxylase 1-like was annotated in *Chenopodium* quinoa Willd (Gene ID: TRINITY_DN47998_c0_g1).

**Figure 7:**
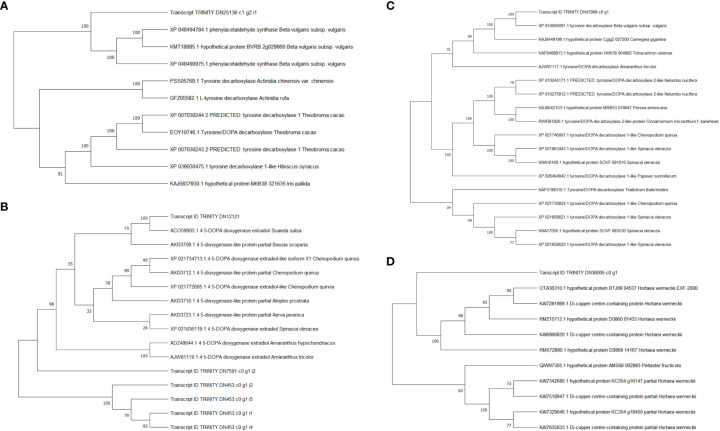
Phylogenetic analysis of betalain biosynthetic pathway DEGs. **(A)** Predicted: tyrosine decarboxylase 1, Transcript ID: TRINITY_DN25136_c1_g2. **(B)** 4,5-DOPA dioxygenase extradiol, Transcript ID: TRINITY_DN12121_c0_g1. **(C)** tyrosine/DOPA decarboxylase 1-like, Transcript ID: TRINITY_DN47998_c0_g1. **(D)** tyrosinase like protein orsC, Transcript ID : TRINITY_DN36005_c0_g1.

Compare the sequences obtained from the experiment with those in the NCBI database, and the phylogenetic analysis results showed reliability 4,5-DOPA dioxygenase extradiol, predicted: tyrosine decarboxylase 1 and tyrosine/DOPA decarboxylase 1-like were 100 (**Figure 7**). However, the reliability of tyrosinase like protein orsC was below 70. The results showed that 4,5-DOPA dioxygenase extradiol, predicted: tyrosine decarboxylase 1 and tyrosine/DOPA decarboxylase 1-like are involved in the regulation of betacyanin biosynthesis in *S. salsa.*

### Expression of key genes in the betacyanin biosynthesis pathway

**Figure 8:**
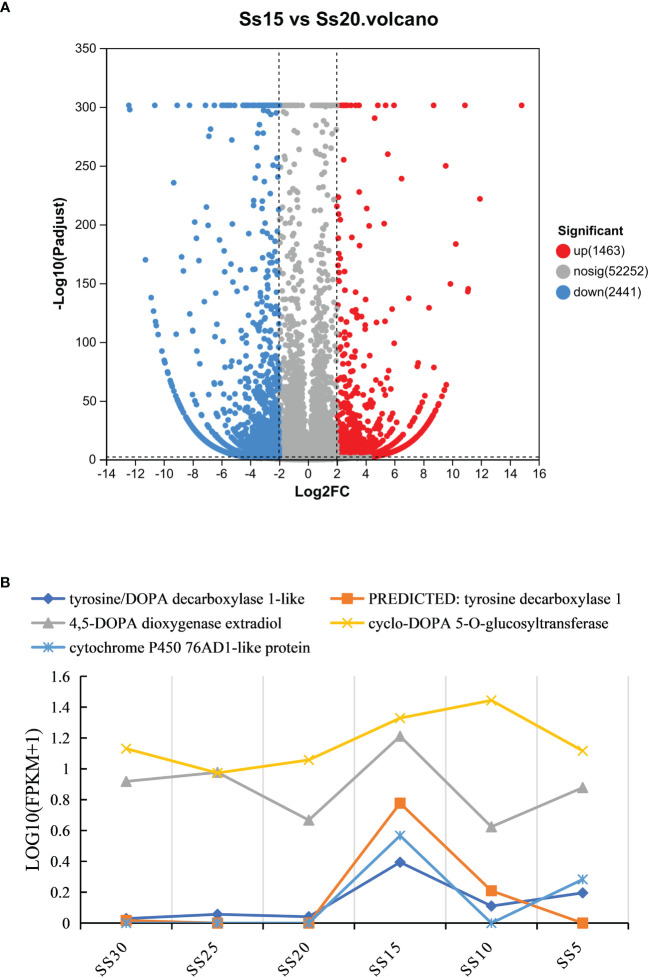
**(A)** Distribution and abundance distribution of up- and downregulated proteins of *S. salsa* in Ss15 vs Ss20. Red and blue indicate up- and downregulated differential proteins, respectively, and gray indicates no significant difference in protein. **(B)** Expression of four key enzyme genes, *TYR*, *DODA*, *UGTs*, and *Cyp450*s under different temperature conditions.

**Figure 9:**
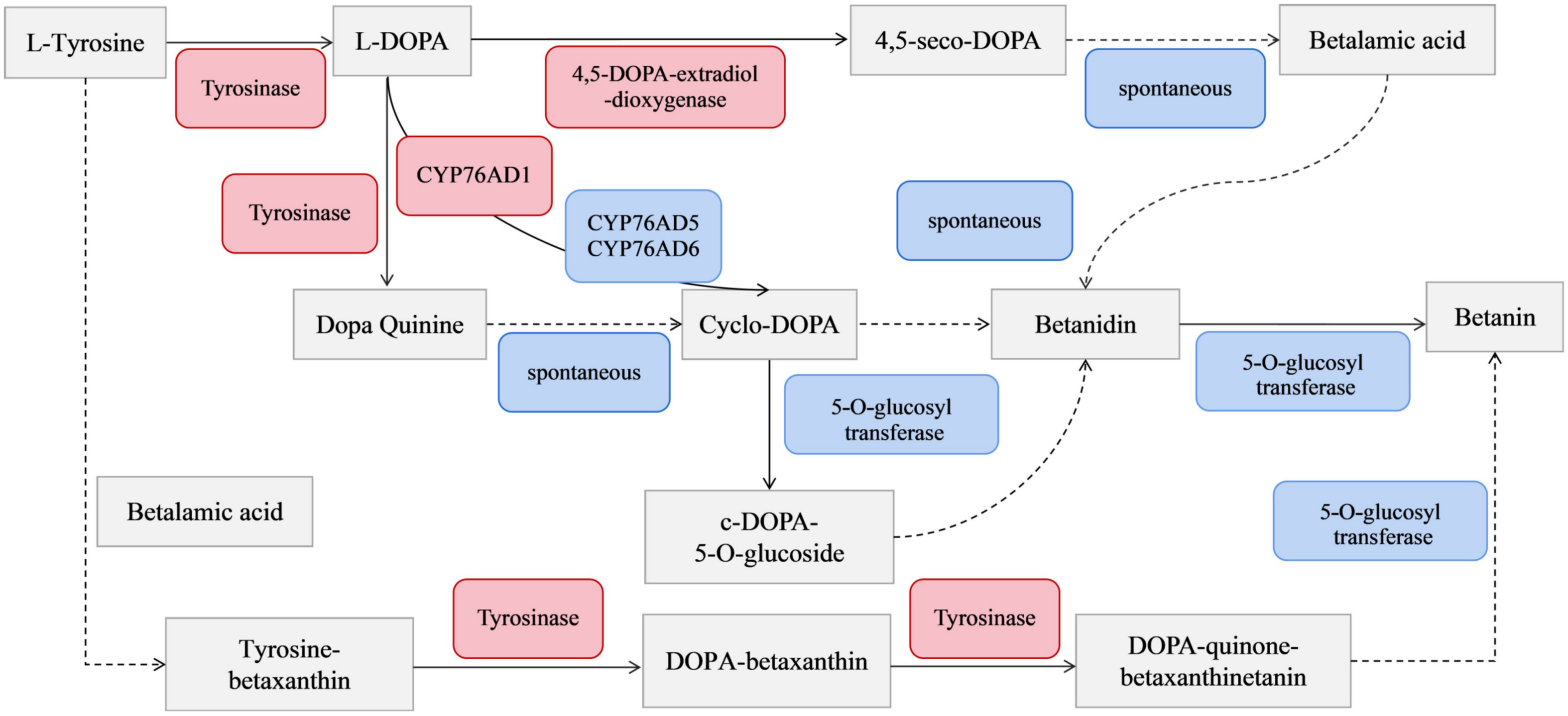
Betacyanin biosynthesis pathway. Red indicates upregulation of genes; blue indicates spontaneous reactions and genes without significant changes (*P <* 0.05). Solid lines indicate reactions involving key enzymes process. The dotted line represents the spontaneous reaction process.

**Figure 10:**
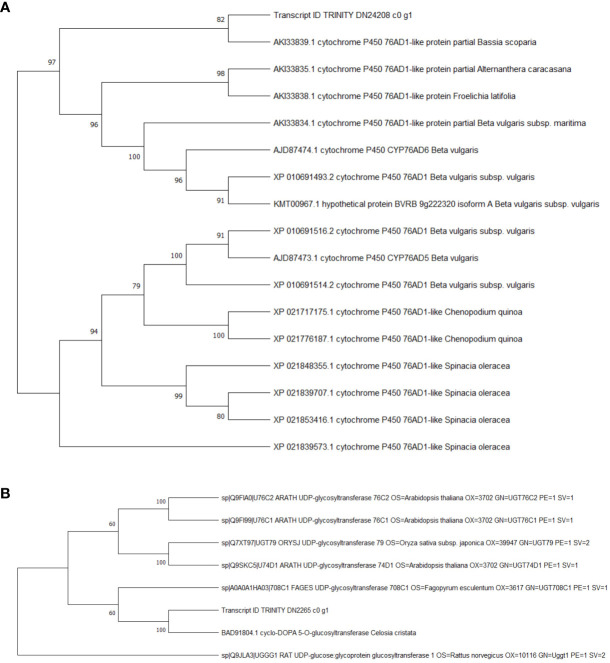
Phylogenetic analysis of Cyp450s and UGTs. **(A)** cyclo-DOPA 5-O-glucosyltransferase, Transcript ID: TRINITY_DN24208_c0_g1. **(B)** cytochrome P450 76AD1-like protein, Transcript ID: TRINITY_DN2265_c0_g1.

The results of KEGG enrichment analysis showed that the DEGs were significantly enriched in the “biosynthesis of betalain” pathway. Further analysis was conducted on the difference groups in the Ss20vsSs15 samples. At 20°C, there were 1463 upregulated and 2441 downregulated DEGs compared to 15°C (**Figure 8A**). Among them, the genes for TYR, Cyp450s, UGTs and DOPA, the key enzymes in the betacyanin synthesis pathway of *S. salsa*, were significantly downregulated, and their expression levels were highest at 15°C. Expression of the gene for 5-O-glucosyltransferase was significantly upregulated at 10°C and significantly higher than for the other five temperature groups (*P* < 0.001, |log_2_FC| ≥ 1) (**Figures 8B**, **9**). At the same, phylogenetic analysis in cyclo-DOPA 5-O-glucosyltransferase and cytochrome P450 76AD1-like protein results showed that which indeed for the betanin biosynthetic pathway (**Figure 10**). Thus, 15°C was conducive to betalain accumulation in *S. salsa* compared to the other five temperatures.

### Key transcription factors regulating betanin synthesis

To further explore the regulatory mechanism of temperature on the synthesis of betanin from *S. salsa*, combined with the results of betacyanin content determination and KEGG enrichment analysis, the TFs of the Ss15vsSs20 differential comparison groups were further analyzed. A total of 597 genes were assigned to 33 TF families in Ss15vsSs20, and the top 20 TF families with significant differential expression were selected (**Table 5**). Compared with the 20°C, a total of 83 TFs were significantly upregulated and 25 TFs were significantly downregulated at 15°C (*P* < 0.001, BH= 2). Among the upregulated TF families, AP2/ERF had the highest number of upregulated genes (15), followed by C2C2 (12) and bHLH (10). At the same time, the number of downregulated genes in the MYB family was the highest (four), followed by MADS (three).

**Table 5:** The TF family and their contained dys-regulated gene number.

TF family	Up gene num	Down gene num.
MYB	9	4
C2C2	12	1
bHLH	10	1
AP2/ERF	15	2
B3_superfamily	3	1
NAC	5	2
WRKY	4	2
bZIP	7	2
GRAS	1	0
C3H	1	0
MADS	2	3
LBD (AS2/LOB)	4	1
FAR1	0	2
SBP	1	2
GRF	3	0
TCP	1	0
C2H2	3	1
LOB	0	1
HSF	1	0
Nin-like	1	0

Up gene num, Up-regulated gene number; Down gene num, Down-regulated gene number.

The MYB, C2C2, bHLH, and AP2/ER TFs regulate the synthesis of betanin in *S. salsa*. The 13 DEGs of MYB were positively correlated with betanin synthesis related genes (e.g. *MYB1R1*, *MYB23*, *MYB14-like*, *CgMYB1*, and *MYB46-like*), while the four DEGs of MYB were negatively correlated with betanin synthesis related genes (e.g. *protein RADIALIS*, *MYB62-like*, *MYB306*, and *MYB108 like*). Among the members of the bHLH family, 10 DEGs were positively correlated with genes related to betanin synthesis (e.g. *bHLH90*, *bHLH93*, *bHLH35*, and *bHLH47*), but negatively correlated with one DEG (*UNE10*). Among AP2/ERF family members, 15 DEGs were positively correlated with genes related to betanin synthesis (e.g. *ERF023*, *dehydrogenation-responsive element binding protein*, *ERF043*, and *ERF061*), but negatively correlated with two DEGs (*PTI5* and *ERF098*). Among members of the C2C2 family, 12 DEGs were positively correlated with genes related to betanin synthesis (e.g. *DOF1.4*, *DOF3.4*, *DOF2.1*, and *DOF2.5*), but negatively correlated with one DEG (*DOF1.5*).

Annotate the transcription factors obtained in this study, and the results indicate that MYB1R1 and CgMYB1 have the function of regulating the synthesis of betaine in *Pyrus* spp (FPKM = 6.61) and *Allium* cepa L. respectively (FPKM = 49.84).

## Discussion

### Effect of temperature on betacyanin content

Temperature will affect stability of the betacyanins of *Monascus* (Abdollahi et al., 2021). Low temperature can induce accumulation of anthocyanins in begonia, which has a protective effect against low temperature stress (Dong et al., 2018). Betacyanins have similar functions to anthocyanins, making plants brightly colored. The accumulation of betacyanins in *S. salsa* leaves has a positive impact on light protection during low temperature stress (Gandia and Garcia, 2019). Betacyanins are effective antioxidants that can significantly contribute to higher thermal stability and tolerance to heat stress of *A. tricolor* photosystem II (Shu et al., 2009). High temperature can significantly inhibit betacyanin synthesis in *B. vulgaris*, and this negative impact is exacerbated on the second or third day of treatment (Ninfali et al., 2017). Lowering temperature is beneficial to betacyanin accumulation in *S. salsa* leaves, possibly related to accelerated chlorophyll decomposition in leaves under low-temperature conditions (Slimen et al., 2017). In this study, compared to 5°C, 10°C, 20°C, 25°C, and 30°C, the betacyanin content in *S. salsa* leaves was significantly higher at 15°C. Below 15°C, the betacyanin content in *S. salsa* leaves gradually increased with decreased temperature, whereas above 15°C, it gradually decreased with increased temperature. This indicates that low temperature is beneficial, and high temperature can significantly inhibit betacyanin synthesis in *S. salsa* leaves – consistent with the results of Wang (2007).

The GO enrichment results showed that the DEGs in Ss15vsSs20 and other four analysis groups were significantly enriched in processes such as “defense response,” “oxidoreductase activity,” Meanwhile, compared with other temperature groups, these terms enriched more up-regulation genes than down-regulation genes in 15°C. The results indicate that compared to other temperatures, the low temperature of 15°C was the most suitable for inducing the accumulation of betacyanins in *S. salsa* leaves. And the biosynthesis of betacyanins may improve the stress resistance of *S. salsa.*

### Effect of temperature on key enzymes of betacyanin synthesis

Dark and light conditions are important factors that affect tyrosinase activity. Tyrosinase can only be detected in *S. salsa* seedlings cultivated in dark conditions and not light conditions. When *S. salsa* seedlings were transferred from darkness to light conditions, the betacyanin content and tyrosinase activity in leaves decreased (Wang, 2007). Tyrosinase activity is also affected by light quality. Under blue light conditions, the blue light receptor anthocyanin 2 (CRY2) of *S. salsa* undergoes protein phosphorylation, resulting in decreased tyrosinase activity, and so decreased betacyanin content. Moreover, accumulation and degradation of CRY2 protein during the light–dark cycle is an irreversible process (Wang, 2007). Ruan (2008) cloned the promoter *Ss DODA* of *DOD* from the *S. salsa* genome and found that the promoter contains an LTR (CCGAAA). It is speculated that low temperature can affect *Ss DODA* expression, thereby regulating betanin metabolism in *S. salsa*. Wang (2011) further explored the effects of temperature on expression of the *Ss DODA* promoter and the betacyanin content in *S. salsa* seedlings. The expression of *Ss DODA* and the betacyanin content significantly increased after 7 days of low-temperature treatment compared to the control group, but then showed a downward trend after 7 days of culture under normal conditions. This indicates that low temperature can affect expression of *Ss DODA* and betacyanin synthesis in *S. salsa*.

In the early spring and deep autumn when temperatures are low, *S. salsa* plants in coastal wetland are red or purplish red, forming a beautiful and spectacular “red beach” landscape on the mudflats, attracting many tourists and creating economic benefits for the local eco-tourism industry. In addition, equipped with antioxidant activity, betacyanin has been widely applied in cosmetic, food additive and pharmaceutical (Siow and Wong, 2016). In this study, tyrosinase was detected under 15°C and light conditions, and the genes for tyrosinase (tyrosine/DOPA decarboxylase 1-like and tyrosine decarboxylase 1), CYP76AD1 (cytochrome P450 76AD1-like protein) and 4,5-DOPA dioxygenase (4,5-DOPA dioxygenase extradiol) were significantly upregulated at this temperature, with the highest expression level. At 10°C, expression of the gene for 5-O-glucosyltransferase (cyclo-DOPA 5-O-glucosyltransferase) was significantly upregulated. Combined with the betacyanin content results, this further demonstrates that 15°C is conducive to increased betacyanin synthesis in *S. salsa*, which is consistent with observations of *S. salsa* under natural conditions. Therefore, it should be possible to conduct regulation experiments with different control factors based on a temperature of 15°C, such as single or composite factor stress studies with different light intensities and salinity. At the same time, combining molecular techniques such as gene silencing or knockout, will allow a deeper exploration of the discoloration and regulatory mechanisms of *S. salsa*, and to induce *S. salsa* to accumulate large amounts of betacyanins, with a view to maximizing the reddening time of *S. salsa*, achieving both ecological protection and economic creation.

### Effect of temperature on TFs and other metabolic pathways

Betacyanin accumulation in *S. salsa* is regulated by TFs. The MYB and bHLH TFs play key roles in various biochemical processes and participate in regulation of anthocyanin biosynthesis (Toledo-Ortiz et al., 2003). The TF HubHLH159 in pitaya promotes betacyanin biosynthesis by activating expression of *HuADH1*, *HuCYP76AD1-1*, and *HuDODA1* (Chen et al., 2023). In Arabidopsis, *AtbHLH132*, *AtbHLH32*, and *AtbHLH101* of the bHLH family participate in regulation of anthocyanin biosynthesis (Bailey et al., 2003). In apples, MdbHLH3 has been identified as a key bHLH TF involved in anthocyanin synthesis (Espley et al., 2007). In this study, 11 DEGs were annotated in the bHLH TF family, and 10 TF genes including *bHLH93* and *bHLH35* were significantly upregulated (*P* < 0.001); 13 DEGs were annotated into the MYB TF family, and nine TF genes including *MYB1R1, MYB1* and *MYB14* were significantly upregulated (*P* < 0.001). Gene *bHLH93* can induce plant defense responses and improve plant resistance to stress (Zhou et al., 2020); *bHLH35* can regulate rice anther development (Ortolan et al., 2021). Gene *MYB062* is related to the lignin biosynthesis pathway in flax (Tombuloglu, 2020). Plant stilbene is a plant antitoxin that accumulates in a few plant species including *Vitis vinifera* in response to biotic and abiotic stresses, and has many beneficial effects on human health (Holl et al., 2013). *MYB14* can participate in transcriptional regulation of stilbene biosynthesis in *V. vinifera*, specifically activating the promoter of gene *STS*, and promoting accumulation of glycosylated stilbene in plants. In this study, MYB1R1 TF in Ss20vsSs15 was significantly upregulated (*P* < 0.01). BvMYB1 is a typical plant R2R3-MYB containing the R2 and R3 MYB domains and a C-terminal activation domain, which is a good candidate to regulate betalains (Hatlestad et al., 2015). MYB1R1 is an important regulatory factor involved in plant pigment synthesis. The content of anthocyanins in pears is positively correlated with *MYB1R1* expression (Liu et al., 2019), which may play an important regulatory role in metabolism of carotenoids and can directly or indirectly regulate carotenoid cleavage dioxygenases (Zhang et al., 2019). Therefore, *S. salsa* betacyanin synthesis may be mainly regulated by *MYB1R1 and MYB1* TFs, while *MYB14* may play an important role in resistance response to stress in *S. salsa*. The TFs for *MYB062*, *bHLH159*, *bHLH132*, *bHLH32*, *bHLH101*, and *bHLH3* were not detected in this experiment.

Phenylpropane metabolism is one of the most important secondary metabolic pathways in plants. Flavonoid compounds are the most diverse branch pathways of propane metabolism, and play an important role in plant growth, development, and environmental interactions (Dong and Lin, 2021). Phenylpropane metabolism is influenced by various biological factors and abiotic stresses such as temperature and light, and is regulated by multiple regulatory pathways including MYB. Gene *McMYB4* participates in the abiotic resistance and growth of apples with temperature changes by regulating phenylpropane metabolism and hormone signaling (Hao et al., 2021). Browning of Nanguo pear peel induced by low temperature is related to phenylpropane metabolism (Sun et al., 2022), Glycine betaine can enhance the activity of enzymes related to phenylpropane metabolism, thereby improving the cold resistance of peaches (Wang et al., 2019). Tea shoots appear white-green at low temperatures and turn green as temperature increases. Ma et al. (2018) and others conducted transcriptome analysis to explore the discoloration mechanism of tea shoots. The results showed that the color change of “Huabai 1” buds was caused by the comprehensive action of the phenylpropanoid biosynthesis pathway and other metabolic pathways, including flavonoid biosynthesis. In this study, the DEGs in Ss20vsSs15 were mainly involved in processes such as “phenylpropane metabolism,” “response to stimulation,” “biological regulation,” and “flavonoid biosynthesis.” Therefore, we predict that betacyanin accumulation is comprehensively affected by processes such as “phenylpropane metabolism” and “flavonoid biosynthesis,” which overall improve the stress resistance and antioxidant level of *S. salsa*. This is basically consistent with the research results of Song et al. Red-violet phenotype of *S. salsa* in the intertidal habitat have higher differential proteins involved in defense, stimulation, and stress response than those green phenotype of *S. salsa* in the supratidal habitat, which promoted the biosynthesis of phenylpropanoids and other secondary metabolites, further increased the medicinal value and resisted environmental stresses ability, but directly reduced the palatability of *S. salsa* (Song et al, 2021). *S. salsa* is not only one of the best nature-based restoration solution for shoreline stabilization, but also the important biological barrier of coastal wetlands (He et al., 2022). This provides a theoretical reference for further exploring the discoloration mechanism of *S. salsa.*

## Conclusion

In this study, low temperature (15°C) promoted betanin accumulation in *S. salsa* leaves by upregulating the expression of key structural genes (for tyrosinase, CYP76AD1 and 4,5-DOPA dioxygenase) and related TFs (*MYB1R1* and *MYB1*) involved in betacyanin synthesis. At the same time, the antioxidant activity and peroxidase expression of *S. salsa* leaves in 15°C were upregulated, and the DEGs in Ss20vsSs15 were mainly involved in processes such as “phenylpropane metabolism,” “defense response,” “oxidoreductase activity,” and “flavonoid biosynthesis.” Therefore, we predict that the synthesis of betacyanin enhances the stress resistance and antioxidant level of *S. salsa.* The research results provide reference for adding a “Red Beach” and the restoration of *S. salsa* in the coastal wetlands.

## Data availability statement

The original contributions presented in the study are publicly available. This data can be found here: NCBI, accession: PRJNA955095.

## Author contributions

ML: performed the experiments, completed data analysis and wrote the manuscript. PH: conceived the experimental design, and fund support. ZZ: guided to cultivating plant. JL and HL: modified of the manuscript. SM, YS and BL contributed to sample collection. All authors contributed to the article and approved the submitted version.
